# Multi-Source Diagnosis of Bearing Faults Using Interpretable Boosted Trees

**DOI:** 10.3390/s26051576

**Published:** 2026-03-03

**Authors:** Miguel Fernández-Temprano, Manuel Astorgano-Antón, Óscar Duque-Pérez, Vanesa Fernandez-Cavero, Daniel Morinigo-Sotelo

**Affiliations:** 1 Department of Statistics and Operational Research, Escuela de Ingenierías Industriales, Universidad de Valladolid, 47011 Valladolid, Spain; manuastant@gmail.com; 2 Department of Electrical Engineering, Escuela de Ingenierías Industriales, Universidad de Valladolid, 47011 Valladolid, Spain; oscar.duque@uva.es (Ó.D.-P.); vanesa.fernandez.cavero@uva.es (V.F.-C.); daniel.morinigo@uva.es (D.M.-S.)

**Keywords:** diagnostic expert systems, induction motors, monitoring, boosted trees, SHAP values, data fusion

## Abstract

The early detection and diagnosis of faults in induction motors is vital in today’s industry, since these are the motors used for the largest number of applications in the industrial environment and failure to detect a fault early can lead to significant losses. Bearing faults are the main problems detected in induction motors and several techniques have been developed to detect them. The use of the information contained in the motor vibrations is the main traditional source for its diagnosis, although there are also proposals that use the supply current, or the sound of the motor. Furthermore, these variables can be used in the time domain or in the frequency domain. The purpose of this work is to use explainable artificial intelligence (XAI) to determine which of these variables, and in which domain, contributes most to a correct diagnosis and how much can be gained in diagnosis by using multisensor data fusion. To carry out this comparison in the most objective way possible, a model selection procedure is proposed and boosting techniques are considered that prove to give a very precise diagnosis. The obtained diagnostic rules are then interpreted using SHAP values, a recent interpretation technique for complex classification procedures.

## 1. Introduction

Induction motors are a vital element in today’s industry, as they are used for a wide variety of applications. Induction motors are very robust and reliable; however, they are not free of faults. An undetected fault can cause major and severe failures, which can result in a production stoppage, incurring significant costs. In order to avoid this situation, it is vital to carry out a correct fault diagnosis at an early stage in order to anticipate this type of situation.

One of the most common faults that occur in induction motors is bearing failure [1,2], so it is essential to carry out a correct and precise diagnosis of bearings. If the technical literature is analyzed, it can be seen that the most suitable technique for diagnosing bearings is vibration [3]. However, the use of the motor supply current as a diagnostic variable (technique known as MCSA, Motor Current Signature Analysis) has recognized advantages [3,4], mainly associated with the simplicity and non-invasive nature of the sensors to be used. The use of MCSA as a diagnostic technique for induction motors has already been widely presented in the literature, being recognized as a very useful technique to diagnose problems in the rotor bars and associated with the eccentricity of the rotor [5,6]. Nevertheless, although different proposals can be found in the literature, it is still not recognized as a basic technique for bearing diagnosis [7]. This is mainly due to the fact that the vibrations associated with these defects have a low corresponding energy, which means that their translation to the current implies that the symptoms of failure in the current spectrum may be hidden among the signals noise, hindering diagnostic capability [8]. Acoustic-based procedures have also been proposed [9] with the advantages of low cost and no contact with the equipment [10] although they are more sensitive to noise [11]. Regardless of the technique used, it must also be taken into account that, given the importance mentioned of induction motors and, therefore, of their diagnosis, the reliability and accuracy of such diagnosis is fundamental. The possibility of false positives or false negatives can, in turn, lead to significant costs and loss of confidence in the diagnostic tool used. Several situations that can lead to false diagnoses have been identified in the literature [12,13]. Therefore, one of the main objectives of this work is to analyze the diagnostic capability of different variables (current, vibration and sound) used to monitor the condition of bearings in induction motors. For the purpose of determining which of these variables can provide more information, and in which cases the combination of several of them can be useful for improving the reliability of the diagnosis, a technique known as multisensor (or multisource) data fusion may be used [14,15,16,17,18]. For example, for critical motors, due to their importance within the installation, combined monitoring would be adequate to increase the accuracy of the diagnosis, while in less critical cases, it may be sufficient to monitor only one of the variables. To evaluate the diagnostic capacity of these variables individually or in combination, a machine learning-based classification technique was chosen to establish a fair comparison between the different options. After evaluating different algorithms (as can be seen in the comparison presented in the Conclusions section), it was decided to use boosting [19], as it provided better results and is a very efficient method to reduce errors in predictive data analysis. It can be extended for use in predictive failure analysis, since it improves predictive accuracy and the performance of the models used.

The interpretability of the results obtained from complex techniques, such as boosting, has been considered one of the main drawbacks of these techniques. For this reason, much work has been done in recent years to provide explainability to these so-called black-box models. XAI techniques aim to make these models transparent, understandable and interpretable [20]. The amount of work being developed in this area is so large that there are even reviews for XAI applications in specific fields such as computer vision [21], social sciences [22], networking [23], medicine [24] and industrial fault diagnosis [25]. There are also many comprehensive reviews of XAI such as [26,27,28]. A good guide to XAI is [29], which describes the theoretical foundations and the main techniques for XAI. In [29], XAI techniques for black-box models are classified into Post Hoc Interpretation Techniques, Feature Attribution Methods, Counterfactual Explanations, Causal Inference Techniques, Graph-based Explanation Techniques, and Multimodal Explainability and are described in detail. Since one of the objectives of this work is to identify which variables, and to what extent, are more relevant in our classification procedures, we considered a Feature Attribution Method. Specifically, we consider SHAP [30], which, according to [25], has gained considerable attention from the research community because it uses a more straightforward strategy than other techniques that can be integrated into any black-box machine learning model. In this work, we show how this allows us to identify which variables are most significant in the procedure and to give physical interpretations of the results.

As can be seen in the systematic review in [25], there are not many studies that consider XAI in the bearing fault diagnosis problem we consider here. In addition to the four studies [31,32,33,34] mentioned in [25], we have also found another recent one [35]. To highlight the novelty of the present study, we will now describe the characteristics of these five studies. Unlike the approach taken in this article, none of these five studies considers different sources of information. All of them consider vibration signals, and only one [32] also considers current signals, but it does not combine them with vibration or sound. Only two of them [32,35] employ cross-validation to evaluate the results and avoid overfitting the models. Two of them [34,35] use SHAP as their XAI methodology, and none of them use boosting as a method for constructing classification rules. Four of them [31,32,33,34] consider more complex methods based on different network architectures, while [35] uses a procedure that includes k-NN and Random Forest, procedures that, based on the comparison we develop in the final section of this work, perform worse than the XGBoost method considered here.

The main contributions of this work, from a methodological point of view, are to show the usefulness of boosting as a classification technique for the diagnosis of bearing failures in induction motors, to propose the use of SHAP values to solve one of the main drawbacks of machine learning techniques which is the lack of interpretability of the results [36], thus highlighting the interest in XAI techniques in this field, and to demonstrate the ability of these techniques, when combined, to diagnose progressive deterioration. From a practical application point of view, an analysis is undertaken of the diagnostic capacity provided by the use of different motor operation signals such as stator current, vibrations and sound, comparing also the use in the frequency domain and statistics in the time domain, in order to reach a compromise between reliability, computational cost and practical implications of variable acquisition, focusing on applicability in terms of predictive maintenance.

This paper is organized as follows: First, in Section 2, a workflow scheme is given describing the steps of the procedure considered to solve the diagnostics problem that is being dealt with. Also in this section, the methodology used is analyzed, describing the classification and interpretation techniques used, as well as the data considered. Section 3 considers the test bench used, as well as the tests carried out. Finally, Section 4 presents the results achieved, Section 5 provides a physical interpretation of the XAI results, and Section 6 discusses what can be concluded from them.

## 2. Methodology

**Figure 1:**
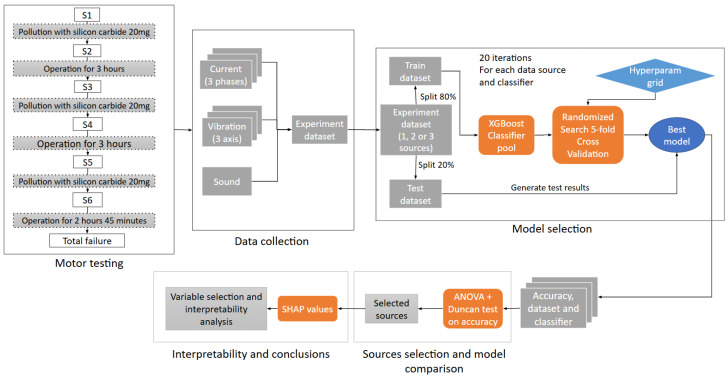
Methodology workflow.

In Figure 1, a detailed scheme of the steps followed to tackle the diagnostic and classification problem is given. The first two blocks deal with the motor testing and the collection of the data from the different sources considered. The methodological details on the data are explained in Section 2.1, while the technical details on the experimentation appear in Section 3.1. Then, as can be seen in the third block of Figure 1, these data are processed using the boosted classification trees developed in Section 2.2 and a model selection procedure detailed in Section 2.3. Finally, the models obtained for the different sources are compared and selected using statistical ANOVA procedures and interpreted considering the recent SHAP values technique explained in Section 2.4.

### 2.1. Fault Signatures

A bearing defect will cause a radial movement between stator and rotor that will modify the air gap of the motor, resulting in bidirectional rotating eccentricities [37] that can be denoted in the physical variables associated with the operation of the motor. Depending on the type of bearing defect (inner or outer race, train defect or balls defect), characteristic vibration frequencies will appear, which are a function of the bearing composition and geometry, as given by (1)–(5) [38]:

(1) $$f_{v}=q\frac{1-s}{p}f_{1},$$

where $f_{v}$ is the vibration fault frequency, $f_{1}$ is the supply frequency, *s* is the slip, *p* is the number of pair of poles and *q* depends on the type of fault, being:

- Fundamental Train Frequency (FTF):
  (2) $$q_{FTF}=\frac{P_{d}}{2B_{d}}\left(1-A^{2}\right).$$
- Ball Pass Frequency Inner Race (BPFI):
  (3) $$q_{BPFI}=\frac{N_{b}}{2}\left(1+A\right).$$
- Ball Pass Frequency Outer Race (BPFO):
  (4) $$q_{BPFO}=\frac{N_{b}}{2}\left(1-A\right).$$
- Ball Spin Frequency (BSF):
  (5) $$q_{BSF}=\frac{1}{2}\left(1-A\right).$$
  where $P_{d}$ is the ball pitch diameter, $B_{d}$ the ball diameter, $N_{b}$ the number of balls, and $A=\frac{B_{d}}{P_{d}}\cos\alpha$, where α is the ball and races contact angle.

The vibrations denoted by the above equations will produce changes in the motor air gap that will result in the generation of harmonics in the motor supply current, at frequencies $f_{brn}$ given by (6)

(6) $$f_{brn}=f_{1}\pm nf_{v}.$$

As discussed in the introduction, to determine the diagnostic capability of different physical variables related to motor operation, information on the stator current, the sound emitted and the vibrations of the motor were collected. Time domain statistical features are considered. These features are commonly used in vibration signal statistical analyses [39,40] and have also been proposed as input in motor current signature analysis [41]. For the three sources of information, thirteen high-order statistics (see Table 1 for their full description) were computed on the corresponding signals. Furthermore, for the current signal, more detailed information on the frequency domain was also registered. To be more precise, the information recorded for these three sources, used as predictors in the classification rules generated, is as follows:

- Current: data about the motor stator current is collected. For the frequency domain, there are 4 variables associated to each type of bearing fault, as given by (2)–(5). These characteristic frequencies are in principle associated with the fundamental component of the signal, but can also be observed around the different harmonics of the signal, by substituting $f_{1}$ in Equation (6) for the frequency of each harmonic to be considered. In this way, more use is made of the information contained in the signal spectrum. Specifically, in this work, the first 11 odd harmonics and 11 sidebands (with plus and minus signs) around them are considered [42]. This results in 4·11·22=969 independent variables for each current phase. Moreover, the 13 high-order statistics for each of the 3 phases (i.e., 39 independent variables on the temporal domain) are also computed.
- Sound: the sound emitted by the motor is recorded, and the corresponding 13 high-order statistics are obtained, as given by Table 1.
- Vibration: the vibration signal is acquired, using an accelerometer, and the 13 high-order statistics given in Table 1 are computed. In this case, the vibration occurs along the three axes (X, Y, Z) and the number of variables is also multiplied by 3.

### 2.2. Boosted Classification Trees

Initially, both neural networks and boosted trees were considered to carry out the experiment. However, only the latter was used due to their difference in performance. Therefore, in order to save space, only boosted trees are described.

CART or Classification And Regression Trees (see [43,44,45]), are one of the most used predictive models when it comes to simplicity and interpretability. They are easily understood as they essentially apply filters to the predictor variables of their inputs and output their classification. However, decision trees are not robust or precise enough when compared to other more modern machine learning algorithms.

With the increase in computing resources, ensemble models have gained prominence. Ensemble models combine many simple weak classifiers, such as two-leaf trees, to obtain a more powerful one. See, for example, [46,47], for a detailed interpretation on how these procedures work and for some recent developments. In this work, we have considered several tree-based ensemble methods such as Random Forest, classical boosting (AdaBoost) [48], Gradient Boosting Classifier (GBC) [49], Light Gradient Boosting Machine (LightGBM) [50] and eXtreme Gradient Boosting (XGBoost) [51]. Other methods in this line, such as CatBoost [52] or Extremely Randomized Trees (ERT) [53], were not considered. CatBoost was not considered, as we have no categorical features in the dataset, which is the main advantage of that method. ERT works in a similar way to Random Forest, and its main advantage is its speed. This is not relevant in our context since XGBoost is fast enough for the detection of the failures considered in our work.

XGBoost, which will be our final selected algorithm, has been established as one of the best modern machine learning algorithms, as it offers an efficient and generalized implementation of gradient boosting. We now describe its main advantages. Among the new characteristics that XGBoost adds to traditional gradient boosting are:

- Parallelization of the training process by scanning the training instances and defining independent partitions that will appear in the tree. These independent partitions or paths are then processed in parallel.
- Tree pruning. By replacing the traditional greedy method, XGBoost builds each tree up to a maximum predefined depth and then prunes the branches with negative loss.
- Regularization. This penalizes complex models by using L1 and L2 regularization.
- Column and row subsampling. Random omission of observations (rows) and attributes (columns) to avoid overfitting.
- Partition search with absent data. In most machine learning problems, it is common to have missing data. XGBoost includes a small sub-algorithm which learns the patterns of missing data by enumerating absent values (both from start to end and vice versa) and predicting in both directions, choosing the best result.
- Weighted quantile sketch. For large datasets, it is not easy to find candidates to split the data. The dataset is split into smaller subsets and the quantiles are computed for each subset to form an approximate histogram. The quantiles are then weighted so that the sum of the weights within each quantile are approximately the same.

### 2.3. Model Selection

To obtain the best possible models, i.e., the boosted classification rules with lowest error rates, and to prevent possible annoying effects such as overfitting or error rate underestimation, these steps are followed:

1. Training and test sets using hold-out. A total of 80% of the initial dataset is used to train the classifier and the remaining 20% to test it.
2. Using the training partition, the best hyperparameters are chosen with 5-fold cross-validation. As hyperparameter search methods, we considered both random search and Bayesian optimization [54]. In random search, hyperparameters are chosen randomly (within reasonable thresholds) until an arbitrary number of iterations have passed, and no improvements in the accuracy have been observed. Bayesian optimization keeps track of past evaluation results to focus the search on the best previous parameter regions, and therefore it is expected to obtain faster and better results. We report the best results obtained with these two methods. The hyperparameters tuned for this model are the learning rate, the number of trees, their depth, and the weights of the leaves. Additionally, regularization parameters such as L1 and L2, column and row subsampling are also included.
3. The best model from the previous step is built and the error rate is estimated over the test set.
4. All the previous steps are repeated 20 times to reduce the variance in the error estimation.

### 2.4. Model Interpretability

It is well known that interpretability is the main price to be paid when more elaborate procedures, such as boosting, are considered for classification. To overcome this black-box problem, in this paper, we use SHAP (SHapley Additive exPlanations), a technique based on Shapley values [55,56,57,58] that gives more general explanations than those obtained by LIME (Local Interpretable Model-agnostic Explanations) [59], which is another method commonly used to interpret complex models [60].

LIME seeks to interpret how an individual observation *x* is classified. The algorithm behind it generates noise around *x* (obtaining new observations $x^{\prime }$) and computes the classification of $x^{\prime }$, allowing us to interpret the region near *x*.

The Shapley values are based on game theory and inform how to distribute the predictions obtained among the different variables (players) appearing in the classification rule (game). The algorithm takes the inputs *x* and evaluates how the output changes when groups (or coalitions) of variables appearing in *x* are added and subtracted.

Full technical details on these techniques can be found in references such as [29,35,61].

As shown in [30], both LIME and Shapley values are particular cases in a more general framework of model interpretation and SHAP combines characteristics of the two methods since, as in LIME, perturbations of the original data are considered, while it starts from an entire sample as the Shapley values do and not from a single observation as LIME does. SHAP turns out to be the optimal solution in that general framework and fulfills some important theoretical properties such as accuracy and consistency. Moreover, according to [35], there are two main advantages of this SHAP-based model explanation. The first is that the interpretation obtained in this way is inspired by a collaborative game theory scenario where the contributions of each feature attributed to the model’s performance are unequal but cooperate with each other [55]. The Shapley value ensures that each feature attribute benefits as much as, or more than, it would from independent performance. The second advantage is that SHAP can provide a unique solution by satisfying local accuracy (precision of explanations at local level), missingness (good performance when features are missing), and consistency (robustness of explanations across similar data points) based on the original model, thus enabling the model’s explainability [30]. Full details and computational feasible approximations for the computation of SHAP can also be found in [30].

## 3. Dataset

### 3.1. Test Bench Description

**Figure 2:**
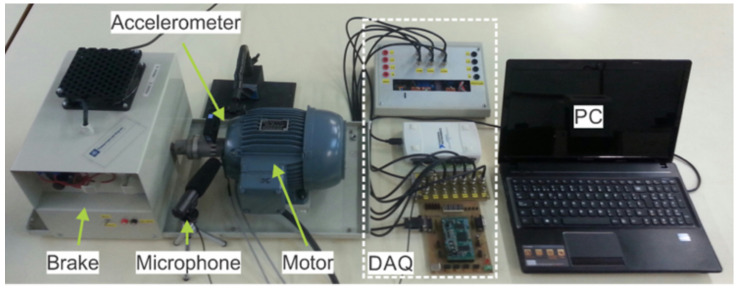
Test bench.

An extensive set of tests was carried out on an induction motor to evaluate its performance under different load conditions. The motor had a rated power of 750 W, two pole pairs, and was fed directly from the line at 50 Hz and loaded with a magnetic powder brake, type 14.512.01.22 from Lenze (Asten, Austria). A NI USB-6210 card (National Instruments, Austin, TX, USA) was used to acquire data during the experiments. In addition, two Hall effect sensors by LEM (Meyrin, Switzerland) were used, one to measure current (LA 25-NP) and one to measure voltage (LV 25-P). During the data acquisition process, the supply voltage, stator current and motor speed were recorded at a sampling rate of 16,384 Hz. Vibration signals were acquired with a triaxial accelerometer LIS331DHL by STMicroelectronics (Geneva, Switzerland), with a range of ±78.5 m/s^2^ at a sampling frequency of 2048 Hz for an acquisition time of 4 s. The sound signal was acquired with a directional microphone model SG-108 from Shenggu (Guangzhou, China) at a sampling frequency of 16,384 Hz for an acquisition time of 4 s. The microphone was placed close to the motor but ensuring that motor vibrations did not interfere with the sound signal. The signals were captured during a steady-state operation of the induction motor. Figure 2 shows the test bench. The chosen sampling frequencies allow achieving a resolution adequate for the purpose of diagnosis, without consuming large resources in terms of acquisition and computation.

**Figure 3:**
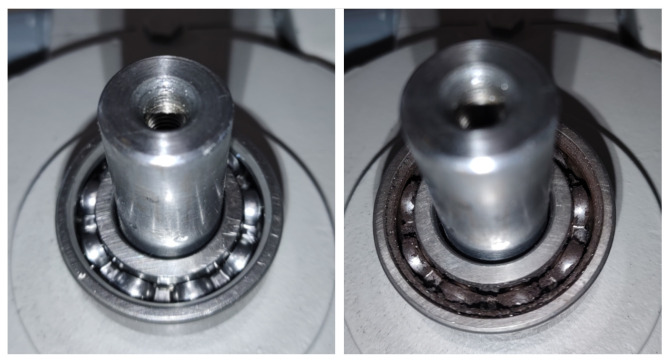
Healthy (**left**, state S1) and complete failure bearings (**right**, state S6).

**Figure 4:**
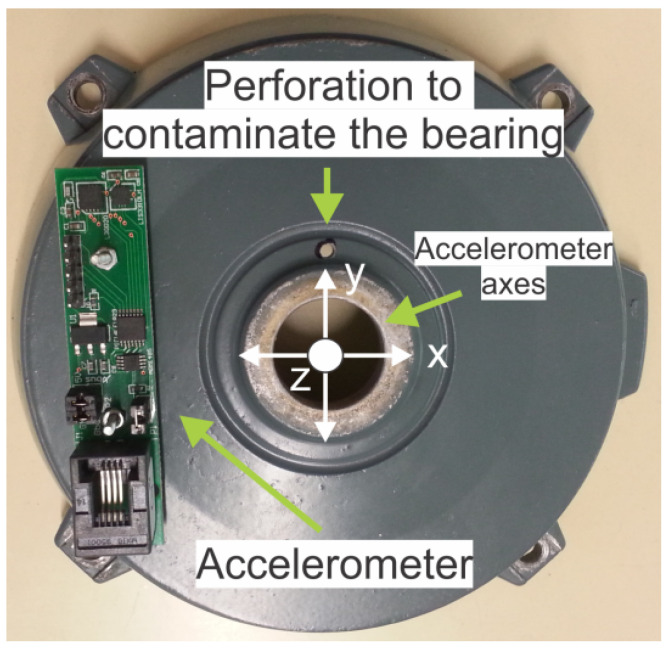
Display of the perforation to contaminate the bearing and the placement of the accelerometer.

The experiments were conducted from the initial state of a new well-functioning, healthy bearing (state S1, Figure 3 left) to the state of complete bearing failure (state S6, Figure 3 right), considering four intermediate stages of wear (states S2, S3, S4 and S5). See Table 2 for a more detailed description of these states. In order to not only analyze the healthy–failure dichotomy but also to be able to determine different states of evolution of the failure, the experiment was designed to obtain six different faulty conditions of the bearing (see first block of Figure 1). For this purpose, the bearing lubricating grease was progressively contaminated with silicon carbide powder through a hole placed in the motor end shield as shown in Figure 4. This material was chosen for its high resistance to erosion and corrosion and high thermal cycling. After performing the tests for the healthy bearing, the grease was contaminated with 20 mg of the abrasive material and the motor was left running for three hours continuously to achieve bearing degradation. The tests for the S2 condition were then performed. This process was repeated five times until the bearing was completely broken; see Figure 3 (right) where all the components of the bearing, inner and outer races, cage and balls can be seen blackened due to the corrosive material introduced. The condition reached by the bearing is representative of situations of excessive use, overloading or lack of lubrication [62], resulting in a distributed-type failure rather than a clearly localized (or single-point) defect in one of the bearing components [63,64].

**Figure 5:**
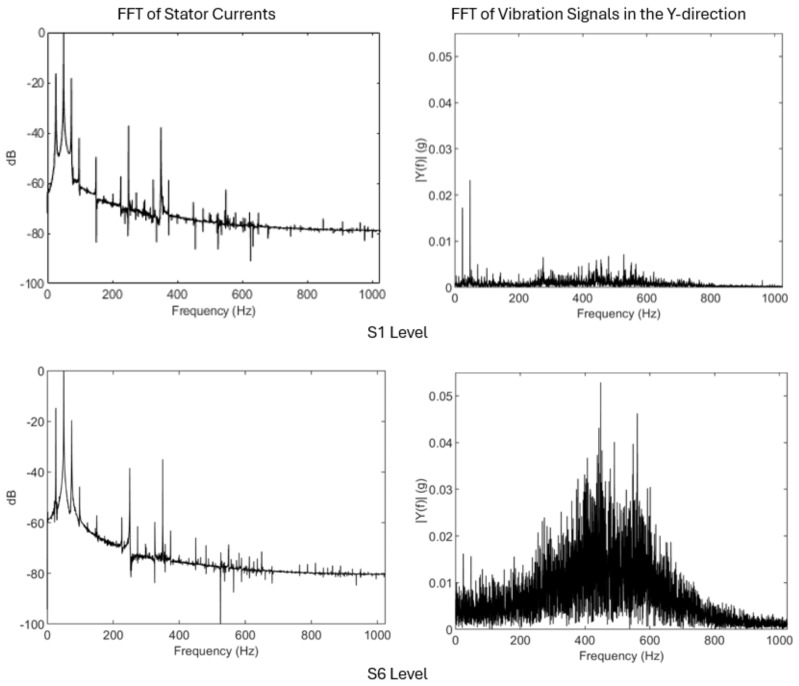
Current and vibrations (y-axis) spectra for the healthy and complete bearing failure states (S1 and S6).

As can be seen in Table 2, from the numerous tests collected, the 60 most representative experiments were selected, with a balanced sample for each failure level, 10 samples for each failure state. In each of these samples, the current signal supplied to the motor, the sound emitted and the vibrations of the motor were recorded, thus providing complete information for each of the states evaluated. Figure 5 shows the spectra of current and vibrations on the y-axis (as most representative) for the initial and final states. It can be seen how there is a clear increase in the energy associated with vibrations from the healthy case to the failure case. However, in the case of current, this increase is not noticeable at first sight, due to the distributed nature of the bearing failure, an aspect that corroborates the decision to use a large number of features associated with the current spectrum.

### 3.2. Preprocessing

As mentioned above, out of the total number of tests performed, we looked for incorrect observations due to measurement failures or lack of precision. No such situation was found for the higher-order statistical values in any of the three different sources mentioned in the previous subsection. However, some such problems were observed for the frequency domain variables. In these cases, since the number of such variables is much larger than the number of tests, predictor variables for which such imprecise values were found were removed, so that the total number of 60 tests is maintained. Of the initial 969 variables, 903 remained.

To keep the dimension of the current frequency dataset under some control, the average of the frequency domain variables of the three phases of the wave was calculated. The statistics-based dataset is widened considering the statistics for the three phases as predictor variables. The same is done with the vibration dataset and its axes.

Since one of the aims of this paper is to investigate the performance of the high-order statistics vs. the frequency domain values in fault classification, we also conducted a principal component analysis [65] and defined two further sets of predictor variables. One of them consisted in the first principal components gathering at least 90% variance, and a second one for which we dropped from the original set of 903 variables those whose correlation with the first principal component was higher than 0.9 (so that they are highly similar to it), thus reducing the number of predictor variables from 903 to 316. The performance of these three different sets of variables in the frequency domain were then compared with that of the high-order statistics set.

## 4. Results

Here, we detail the results obtained for the two questions considered. First, we answer if, for the current data, the information given by the frequency domain data is or not better than that provided by the higher-order statistics for classifying bearing faults. The second question considered is which source of information (current, sound or vibration) is better or if the classification results are improved when combining different sources of information.

All the results appearing in this section have been obtained using Python 3.12.12 and libraries such as Sci-kit Learn, XGBoost and SHAP on a mid-end desktop computer (AMD Ryzen 5 CPU and 16 GB RAM).

Table 3 shows the results obtained using an Analysis of Variance (ANOVA) procedure for comparing the accuracy of the classification models obtained from the current full frequency domain data with those obtained from the higher-order statistics. Notice that the data analyzed in this procedure do not completely fulfill the usual ANOVA assumptions as the values obtained are not independent because the original observations appear in more than one of the cross-validation experiments described in Section 2.3. However, the problem is not as relevant as when serial correlation appears and the F-value in Table 3 is high enough to overcome this problem (notice that the value 298.4 in Table 3 is 76.4 times higher than the value of 4 usually considered for null hypothesis rejection) and shows that there are significant differences among the means. Since the mean error rate of the higher-order statistics model (0.025) is significantly lower than that of the frequency domain data model (0.416), we conclude that the information provided by the higher-order statistics information is better than that provided by the frequency domain data. As mentioned in Section 3.2, the other two configurations were also considered for the frequency domain data. Since worse results were obtained, these results are not detailed here to improve the readability of the paper.

Now, we tackle the second question. As the high-order statistics were shown to perform better for the current data, we compare the rules generated with this information with those generated from the high-order statistics for the sound and vibration sources. We also consider the rules generated from the combination of two of the three sources of information so that we have a total of seven combinations to be compared. The results obtained from the corresponding ANOVA are shown in Table 4.

The very small *p*-values appearing in Table 4 show that there are significant differences among the error rates of the rules generated by the three sources of information and their combinations. As in Table 3, the F-value is high enough (8.18 times higher than the value of 4 usually considered for null hypothesis rejection) to overcome the possible concerns that might be raised by the lack of independence among the error rates obtained in the model selection procedure described in Section 2.3.

In order to know among which rules the differences in accuracy are significant, a post hoc Duncan test [66] was performed. Duncan’s test is a post hoc method widely used in balanced experiments such as the one we are considering here. Among the procedures that can be used for this case, the Duncan test is the least conservative (see [67], page 163). This is the reason why we have considered it. For example, as in Table 5, if the Duncan test does not detect a difference among the results of the current alone set and the current+vibration set, we can be more confident that there is no difference among these groups than if another post hoc method such as Bonferroni or Tukey were considered.

The outputs of Duncan’s test are presented in Table 5, where the sources of information labeled with the same letter in the last column show not significantly different similar error rates. We can see that the sound and vibration sets alone show significantly worse accuracy. Another interesting observation is that the current accuracy results are better (although not significantly better) than those of sound+vibration. Finally, it can be seen that the results obtained when all sources are considered are equal to those obtained with current+vibrations only. Nevertheless, these results are not significantly better than those of current alone. The Duncan procedure was performed under the usual α=0.05 level. We also conducted the procedure under higher α levels and found that under α=0.25, the current and all sources results are still not significantly different, which means that for the equality of these two values to be rejected, we need to assume a type I error higher than 25%.

Now, we present more detailed results for the two most interesting classification procedures obtained, namely, one coming from the current high-order statistics alone and that coming from the fused information obtained from current and vibration sources. Table 6 and Table 7 show the row conditional confusion matrices for the rules obtained with the current and current and vibration sources datasets, respectively. In other words, each row shows the rate of observations of that state that were classified correctly. For example, in Table 6, it can be seen that all observations considered in the procedure described in Section 2.3 coming from class S1 (healthy motors) were classified in the correct class, while 97.5% of the observations coming from class S4 were correctly classified in that class and the other 2.5% observations were incorrectly classified in class S6. Both confusion matrices show the very good performance of these rules.

**Figure 6:**
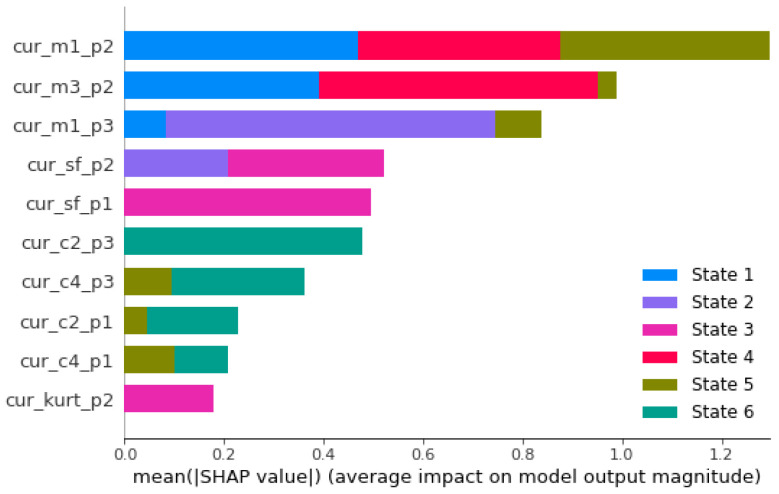
SHAP plots for the 10 most influential features in the current classification rule.

**Figure 7:**
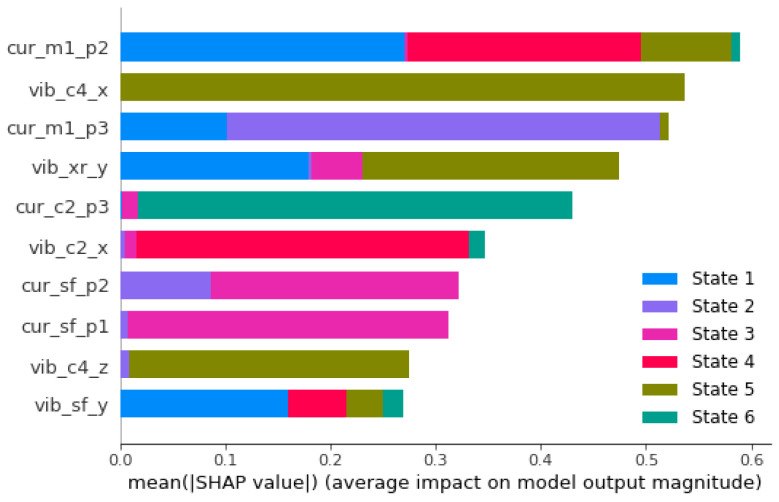
SHAP plots for the 10 most influential features in the current+vibration rule.

Figure 6 and Figure 7 show the SHAP values, described in Section 2.4, for the current and current+vibration classification rules, respectively. In these graphs, the 10 variables that obtained the highest SHAP values in the rules are shown. For each variable in the graph, the first three letters indicate which source the variable comes from (cur for current, vib for vibration), the next letters tell which high-order statistics are considered (according to Table 1) and the final letters indicate, for the current variables, which phase (p1, p2, p3) or, for the vibration variables, which axis (x, y or z) is being considered. The colors in each bar indicate the states for which the corresponding variable is relevant in the classification procedure. For example, we can see in Figure 6 that when the current-only classification procedure is considered, the $m_{1}$ (mean) of phase 2 is the most influential feature and is relevant for classification in states 1, 4 and 5.

## 5. Physical Interpretation of XAI Results

Globally, as shown in Figure 6, the most influential features of the current-only procedure have clear physical significance. The means of phases 2 and 3 (cur_m1_p2, cur_m1_p3) capture electromagnetic asymmetries caused by bearing-induced eccentricities, while the third moment of phase 2 (cur_m3_p2) quantifies the directional bias in current modulations. The presence of 6 out of the 13 high-order statistics among the 10 most influential features indicates that bearing faults create complex signal modifications that require multiple statistical perspectives for complete characterization.

Figure 7 shows the most influential features of the current + vibration classification procedure, revealing the complementary nature of multi-source diagnosis. In this case, 5 of the 10 features come from the vibration dataset, with vibration features (vib_c4_x, vib_c4_z, vib_xr_y) showing notable relevance for advanced fault states (S2, S3, S6). The stability of current features across both single-source and multi-source models (cur_m1_p2, cur_m1_p3, cur_sf_p1, cur_sf_p2) demonstrates the fundamental electromagnetic coupling between bearing faults and current signatures.

While the lowest-order statistic (mean m1) dominates the importance of current features, vibration features are exclusively higher-order statistics (c2, c4, xr, sf). This distinction reflects different coupling mechanisms: current signals show mean value increases due to electromagnetic asymmetries, while vibration signals exhibit impulsive content, requiring higher-order statistical characterization.

SHAP analysis brings out patterns that align directly with the underlying physics of bearing-fault mechanisms. Understanding these physical relationships builds confidence in the diagnostic system and enables informed decision-making in industrial applications.

The prevalence of current-based features in the SHAP rankings (Figure 6) reflects the electromagnetic coupling between mechanical bearing faults and electrical signatures. The most significant features have clear physical significance:

- Mean Value (m1): The high importance of cur_m1_p2 and cur_m1_p3 for states S1, S4, and S5 indicates that bearing faults create dynamic eccentricity, producing spatially asymmetric magnetic field distributions. The resulting time-varying inductance generates spectral modulation in the stator current, appearing as additional harmonic components and modulation of amplitude around the fundamental frequency. Signal processing techniques that compute mean values over analysis windows capture these modulation effects as apparent increases in low-frequency content, providing sensitive indicators of fault progression. This phenomenon is pronounced in the intermediate and advanced stages of the fault, where mechanical asymmetries become considerable.
- Third Moment (m3): The prominence of cur_m3_p2 for states S2, S3, and S5 captures the asymmetric nature of fault-induced current modulations. Bearing defects introduce a directional bias in electromagnetic forces, skewing current distributions quantified by the third moment.
- Shape Factor (sf): The consistent appearance of cur_sf_p1 and cur_sf_p2 across multiple conditions indicates changes in waveform characteristics as bearing faults develop. The shape factor, defined as the ratio of the *RMS* to the mean absolute value, is sensitive to the harmonic distortion introduced by air gap variations induced by the bearing.
- Variance and Fourth Cumulant (c2, c4): These higher-order moments capture changes in the energy distribution and impulsive content of the current signals. The fourth cumulant (c4) is sensitive to the periodic impacts characteristic of bearing faults, whereas variance (c2) quantifies the overall changes in signal energy.

The vibration features identified by SHAP analysis (Figure 7) demonstrate the direct mechanical nature of bearing fault signatures:

- Fourth Cumulant (c4): The high importance of vib_c4_x and vib_c4_z for states S2, S3 and S6 directly reflects the impulsive nature of bearing fault vibrations. Unlike current signals, vibration measurements capture mechanical impact events directly, making the fourth cumulant a robust indicator of fault severity.
- Square Root Value (xr): The significance of vib_xr_y represents the overall energy content of the vibration signal. As bearing faults progress, mechanical impacts increase the signal energy, which is captured in this statistic.
- Variance (c2): Like current signals, vibration variance quantifies the energy distribution, but with direct mechanical coupling to the fault mechanisms.

The SHAP analysis reveals that current and vibration features provide complementary information:

- Current features (cur_m1_p2, cur_m1_p3) dominate for states S1, S4 and S5, indicating their sensitivity to electromagnetic effects of bearing asymmetries.
- Vibration features (vib_c4_x, vib_c4_z) are most important for states S2, S3 and S6, showing their direct coupling to mechanical fault mechanisms

This complementarity explains the improved diagnostic performance when combining current and vibration data, as each modality identifies different aspects of the fault physics.

The SHAP analysis reveals distinct patterns in the features that match the physical progression of bearing degradation:

- Early Stages (S1–S2): Current mean values (cur_m1_p2, cur_m1_p3) show high importance, as initial bearing wear manifests primarily as subtle electromagnetic asymmetries, but mechanical impacts are lower.
- Intermediate Stages (S3–S4): Higher-order current statistics (cur_m3_p2, cur_c4_p3) gain importance, as they capture increased harmonic distortion produced by more pronounced bearing defects. Fault signatures transition from simple mean value increments to complex waveform modifications.
- Advanced Stages (S5–S6): Vibration features (vib_c4_x, vib_c4_z) become dominant, indicating that mechanical impacts now overshadow electromagnetic effects. Direct mechanical coupling provides stronger diagnostic signatures than indirect electromagnetic coupling through current analysis.

This analysis shows that current monitoring is best for incipient faults, while vibration monitoring is key for detecting more advanced faults.

## 6. Discussion and Conclusions

This paper contains several interesting results that we believe generate valuable knowledge. From a methodological point of view, this work exposes the potential value of a cutting-edge classification method, such as boosting, in the detection and classification of bearing faults in a difficult situation, since six different states were considered for the bearing. To reinforce this conclusion, we performed a comparison among different classification methods. We considered not only classical techniques such as Linear Discriminant Analysis (LDA), k-Nearest Neighbours (KNN) and Decision Trees (DT), but also more recent ones such as Support Vector Machines (SVM), Random Forest (RF), AdaBoost, Gradient Boosting Classifier (GBC), Light Gradient Boosting Machine (LGB) and Neural Networks, such as, Multilayer Perceptron (MLP) and 1D-CNN with attention mechanism (CNN-att). The results appear in Table 8. It can be seen that none of these methods perform better than the XGB technique we are considering. The best results for one, two and all three source situations are marked in bold in the table and they all correspond to the XGB procedure. Furthermore, it has been shown that SHAP values allow us to overcome the main drawback of boosting, that of lack of interpretability. It is described how SHAP values allow interpretion of the classification procedures yielding which variables are more relevant for classification and for which classes each variable is relevant. The proposed methodology could be applied to any sector of industry where there are quantifiable variables that denote a deterioration in equipment or system performance.

In terms of efficiency, the XGBoost model required 3416 s for hyperparameter optimization, whereas the CNN-att training took approximately 7887 s (2.3 times longer). Furthermore, the inference time for XGBoost is negligible (0 to 0.1 s per prediction batch), compared to 1 to 2 s per batch for the CNN-att. The minimal inference latency of XGBoost is particularly advantageous in production environments, where on-premise infrastructure is often prioritized to ensure high speed, computational efficiency and system reliability.

From the practical and industrial point of view, relevant result have also been obtained. The first of these is the conclusion that high-order statistics yield better classification results than frequency domain data when only current data are considered. This is interesting because high-order statistics are more easily treated than the frequency domain data.

The second relevant conclusion is that the use of high-order current statistics alone gives better results than considering other single sources of information (sound or vibrations) and that an improvement, although a statistically non-significant one, can be obtained when current and vibrations data are jointly considered as data sources. The fact that obtaining the current data is easier, less invasive, and, in contrast to vibrations and sound, there is no influence of where the sensors are positioned, strengthens the importance of this result.

The XAI analysis provides several practical implications for industrial condition-monitoring systems:

- Sensor Selection Strategy: The SHAP analysis demonstrates that current sensors alone can provide effective early fault detection (states S1–S4). In contrast, the addition of vibration sensors significantly improves the detection of advanced faults (states S5–S6). This finding supports a tiered monitoring approach in which critical motors would employ multi-sensor systems, while less critical applications would rely solely on current monitoring.
- Feature Engineering Guidance: Identifying specific high-order statistics (m1, m3, c2, c4, sf) as the most influential provides clear guidance for feature engineering in industrial implementations. Rather than computing all possible statistical features, systems can focus on these physically meaningful parameters, reducing computational overhead while maintaining diagnostic accuracy.
- Interpretable Decision Support: The SHAP values enable the development of interpretable diagnostic reports that explain why specific fault classifications were made. For example, a diagnosis of state S4 can be explained by pointing to elevated cur_m1_p2 values indicating electromagnetic asymmetry, building operator confidence in automated decisions.
- Maintenance Planning: The fault progression patterns revealed by XAI analysis enable more informed maintenance scheduling. Early detection through current monitoring enables planned maintenance windows, while vibration-based advanced fault detection triggers immediate intervention.

There are several research lines open to be pursued from this work. Notice that the results described here have been obtained under the steady state and with direct supply. It would be interesting to know if the same sort of conclusions can be obtained under the transitory state or if the use of inverters makes a difference in the detection of the faults. This will be a part of our future research.

## Figures and Tables

**Table 1:** High-order statistics.

High-Order Statistic	Formula
Mean (First cumulant)	$m_{1}=\frac{1}{n}\sum_{i=1}^{n}x_{i}$
Second moment	$m_{2}=\frac{1}{n}\sum_{i=1}^{n}x_{i}^{2}$
Third moment	$m_{3}=\frac{1}{n}\sum_{i=1}^{n}x_{i}^{3}$
Fourth moment	$m_{4}=\frac{1}{n}\sum_{i=1}^{n}x_{i}^{4}$
Second cumulant (Variance)	$c_{2}=m_{2}-m_{1}^{2}$
Third cumulant	$c_{3}=m_{3}-3m_{1}m_{2}+2m_{1}^{3}$
Fourth cumulant	$c_{4}=m_{4}-4m_{3}m_{1}-3m_{2}^{2}$
	$\;\;\;\;+12m_{2}m_{1}^{2}-6m_{1}^{4}$
Skewness	$skew=\frac{c_{3}}{{\left(\sqrt{c_{2}}\right)}^{3}}$
Kurtosis	$kurt=\frac{c_{4}+3c_{2}^{2}}{{\left(\sqrt{c_{2}}\right)}^{4}}$
Maximum peak value	$x_{p}=\max\left|x_{i}\right|$
Square root value	$x_{r}={\left(\frac{1}{n}\sum_{i=1}^{n}\sqrt{|x_{i}|}\right)}^{2}$
Crest factor	$cf=\frac{x_{p}}{\sqrt{m_{2}}}$
Shape factor	$sf=\frac{\sqrt{m_{2}}}{\frac{1}{n}\sum_{i=1}^{n}\left|x_{i}\right|}$

**Table 2:** Description of the analyzed bearing conditions and number of experiments for each state.

Bearing State	S1	S2	S3	S4	S5	S6
Description	Healthy bearing	Initial deterioration	Intermediate deterioration	Advanced deterioration	Almost complete deterioration	Complete failure
Experiments	10	10	10	10	10	10

**Table 3:** ANOVA results for the comparison among the accuracy of frequency domain and higher-order statistics models.

	Df	Sum Sq	Mean Sq	F Val	*p*-Value
Variables	1	3.403	3.403	298.4	0
Residuals	38	0.433	0.011		

**Table 4:** ANOVA results for the comparison among the accuracy of the rules obtained with the 3 different sources of information.

	Df	Sum Sq	Mean Sq	F Val	*p*-Value
Dataset	6	0.6878	0.1146	32.72	0
Residuals	133	0.4660	0.0035		

**Table 5:** Post hoc Duncan analysis showing the differences among the accuracy of the rules obtained with the three different sources of information.

	Error	Groups
Sound	0.2125	a
Vibration	0.0700	b
Current	0.0250	c
Sound + Vibration	0.0291	c
Current + Sound	0.0125	c
Current + Vibration	0.0042	c
All three	0.0042	c

Alpha: 0.05. Each group shows similar error rates.

**Table 6:** Row conditional confusion matrix for the rule built for the current high-order statistics dataset and, on the table footer, XGBoost hyperparameter configuration (official API (https://xgboost.readthedocs.io/en/stable/parameter.html, accessed on 1 December 2025)) for the rule. S1–S6 stands for the different states of bearing wear.

		Predicted					
		S1	S2	S3	S4	S5	S6
**Real**	**S1**	1	0	0	0	0	0
	**S2**	0	1	0	0	0	0
	**S3**	0	0	0.925	0	0	0.075
	**S4**	0	0	0	0.975	0	0.025
	**S5**	0.025	0	0	0	0.975	0
	**S6**	0	0.025	0	0	0	0.975

“n_estimators”: 100, “objective”: “multi:softprob”, “learning_rate”: 0.31, “gamma”: 0.0, “subsample”: 0.6, “colsample_bytree”: 0.6, “reg_alpha”: 0, “reg_lambda”: 2.

**Table 7:** Row conditional confusion matrix for the rule built for the current and vibration high-order statistics dataset and, on the table footer, the XGBoost hyperparameter configuration (official API (https://xgboost.readthedocs.io/en/stable/parameter.html, accessed on 1 December 2025)) for the rule. S1–S6 stands for the different states of bearing wear.

		Predicted					
		S1	S2	S3	S4	S5	S6
**Real**	**S1**	1	0	0	0	0	0
	**S2**	0	0.975	0	0.025	0	0
	**S3**	0	0	1	0	0	0
	**S4**	0	0	0	1	0	0
	**S5**	0	0	0	0	1	0
	**S6**	0	0	0	0	0	1

“n_estimators”: 1000, “objective”: “multi:softprob”, “learning_rate”: 0.11, “gamma”: 0.1, “subsample”: 0.7, “colsample_bytree”: 0.9, “reg_alpha”: 0.1, “reg_lambda”: 0.1.

**Table 8:** Error rate of each data source and classification algorithm using both random and Bayesian hyperparameter search. S stands for Sound, V for Vibrations and C for Current. Best results for one, two and all three sources appear in bold.

Method	Sources						
	S	V	C	SV	CS	CV	CVS
**LDA**	0.258	0.141	0.058	0.133	0.091	0.037	0.033
**kNN**	0.354	0.154	0.333	0.154	0.312	0.154	0.154
**DT**	0.213	0.117	0.070	0.083	0.079	0.087	0.091
**SVM**	0.279	0.146	0.345	0.146	0.245	0.146	0.146
**RF**	0.179	0.045	0.029	0.016	0.016	0.016	0.016
**AdaBoost**	0.541	0.466	0.454	0.612	0.083	0.325	0.325
**GBC**	0.183	0.067	0.029	0.029	0.017	0.033	0.025
**LGB**	0.670	0.687	0.604	0.708	0.654	0.608	0.641
**MLP**	0.333	0.063	0.050	0.033	0.033	0.025	0.025
**CNN-att**	0.337	0.075	0.045	0.025	0.029	0.033	0.008
**XGB**	0.212	0.070	**0.025**	0.029	0.012	**0.004**	**0.004**

## Data Availability

The data can be obtained from the authors on request.
